# Optimization Study on Specific Loss Power in Superparamagnetic Hyperthermia with Magnetite Nanoparticles for High Efficiency in Alternative Cancer Therapy

**DOI:** 10.3390/nano11010040

**Published:** 2020-12-26

**Authors:** Costica Caizer

**Affiliations:** Department of Physics, Faculty of Physics, West University of Timisoara, Bv. V. Parvan No 4, 300223 Timisoara, Romania; costica.caizer@e-uvt.ro

**Keywords:** superparamagnetic hyperthermia, magnetite nanoparticles, specific loss power, heating temperature, optimization

## Abstract

The cancer therapy with the lowest possible toxicity is today an issue that raises major difficulties in treating malignant tumors because chemo- and radiotherapy currently used in this field have a high degree of toxicity and in many cases are ineffective. Therefore, alternative solutions are rapidly being sought in cancer therapy, in order to increase efficacy and a reduce or even eliminate toxicity to the body. One of the alternative methods that researchers believe may be the method of the future in cancer therapy is superparamagnetic hyperthermia (SPMHT), because it can be effective in completely destroying tumors while maintaining low toxicity or even without toxicity on the healthy tissues. Superparamagnetic hyperthermia uses the natural thermal effect in the destruction of cancer cells, obtained as a result of the phenomenon of superparamagnetic relaxation of the magnetic nanoparticles (SPMNPs) introduced into the tumor; SPMNPs can heat the cancer cells to 42–43 °C under the action of an external alternating magnetic field with frequency in the range of hundreds of kHz. However, the effectiveness of this alternative method depends very much on finding the optimal conditions in which this method must be applied during the treatment of cancer. In addition to the type of magnetic nanoparticles and the biocompatibility with the biological tissue or nanoparticles biofunctionalization that must be appropriate for the intended purpose a key parameter is the size of the nanoparticles. Also, establishing the appropriate parameters for the external alternating magnetic field (AMF), respectively the amplitude and frequency of the magnetic field are very important in the efficiency and effectiveness of the magnetic hyperthermia method. This paper presents a 3D computational study on specific loss power ($P_{s}$) and heating temperature (ΔT) which allows establishing the optimal conditions that lead to efficient heating of Fe_3_O_4_ nanoparticles, which were found to be the most suitable for use in superparamagnetic hyperthermia (SPMHT), as a non-invasive and alternative technique to chemo- and radiotherapy. The size (diameter) of the nanoparticles (D), the amplitude of the magnetic field (H) and the frequency (f) of AMF were established in order to obtain maximum efficiency in SPMHT and rapid heating of magnetic nanoparticles at the required temperature of 42–43 °C for irreversible destruction of tumors, without affecting healthy tissues. Also, an analysis on the amplitude of the AMF is presented, and how its amplitude influences the power loss and, implicitly, the heating temperature, observables necessary in SPMHT for the efficient destruction of tumor cells. Following our 3D study, we found for Fe_3_O_4_ nanoparticles the optimal diameter of ~16 nm, the optimal range for the amplitude of the magnetic field of 10–25 kA/m and the optimal frequency within the biologically permissible limit in the range of 200–500 kHz. Under the optimal conditions determined for the nanoparticle diameter of 16.3 nm, the magnetic field of 15 kA/m and the frequency of 334 kHz, the magnetite nanoparticles can be quickly heated to obtain the maximum hyperthermic effect on the tumor cells: in only 4.1–4.3 s the temperature reaches 42–43 °C, required in magnetic hyperthermia, with major benefits in practical application in vitro and in vivo, and later in clinical trials.

## 1. Introduction

At present, the most frequently used techniques in cancer therapy are the well-known chemo- and radiotherapy. However, these techniques have a high level of toxicity on the body and in the case of more advanced tumors may even prove ineffective. Therefore, given the high toxicity of current techniques used for treating cancer and the increasing incidence of cancer cases affecting the population [1], it is a necessity to quickly find an alternative method of treatment that proves more effective while less toxic, or, even nontoxic, and which does not effect the healthy tissues.

Magnetic hyperthermia (MHT) using magnetic nanoparticles for cancer therapy is one of the alternative methods to classical chemo- and radiotherapy techniques, which looks to be the most promising in the field due to its non-invasive and low-toxicity characteristics when treating malignant tumors [2,3,4,5,6,7,8,9,10,11,12,13,14,15,16,17,18,19,20,21,22,23,24,25,26,27,28,29]. However, recently, the superparamagnetic hyperthermia (SPMHT) based on the Néel–Brown magnetic relaxation phenomenon [30,31] in superparamagnetic nanoparticles (SPMNPs) [32] is seen as a better alternative in this field, due to the fact that a higher heating power of nanoparticles can be obtained than in MHT which is based on the phenomenon of magnetic hysteresis [2,33].

The most suitable nanoparticles for this type of therapy are the ferrimagnetic ones, spinel type, X^+2^Y_2_^+3^O_4_^−2^ where X is generally a bivalent metal ion (Fe, Ni, Co, Mg etc.) and Y is trivalent Fe [34,35]. However, although there are many studies on ferrimagnetic nanoparticles [36,37,38,39,40,41,42,43,44,45], it has been found that magnetite (Fe_3_O_4_) with the structure Fe^+2^Fe_2_^+3^O_4_^−2^,and having the magnetic structure of inverse spinel Fe^+3^[Fe^+2^Fe^+3^]O_4_^−2^, where the iron ions in the right parenthesis are found in the octahedral lattice and the Fe^+3^ ions outside the parentheses are found in the tetrahedral lattice, is the most suitable for supraparamagnetic hyperthermia [3,4,5,6,7,8,9,10,11,12,13,14,26,29,46,47,48,49,50,51,52,53,54,55]. This is because small magnetite nanoparticles (in nanometers range) in low concentrations (<0.2–0.5 mg/mL) are non-toxic [56]. Also, magnetite nanoparticles exist naturally, being produced by various microorganisms, such as the magnetotactic bacterium [57], the nanoparticles being found in magnetosomes [18,58,59,60]. Thus, these naturally magnetite nanoparticles are also biocompatible and without toxicity. In addition to these aspects, the high-performance magnetic properties of magnetite (high saturation magnetization, low magnetic anisotropy, high magnetic susceptibility, etc.) [34] make it the most suitable to be used in obtaining the hyperthermic effect by superparamagnetic relaxation in SPMHT. For larger nanoparticles, and/or in high concentrations (>0.5 mg/mL) they need to be made biocompatible, by coating them with different organic agents biocompatible with the biological environment where they are to be dispersed [61,62], or by applying bioencapsulation, bioconjugation, etc., to avoid the possible toxicity that would have occurred in the absence of this organic layer. The presence of the organic layer on the surface of the nanoparticles also has the advantage of eliminating the dipole magnetic interactions between the magnetic nanoparticles, with a beneficial effect on superparamagnetic hyperthermia. In addition, when magnetic nanoparticles are coated with an organic layer, for their biocompatibility or functionalization, the thickness of this layer and the resulting hydrodynamic diameter must be taken into account, which can sometimes influence the specific power dissipated in magnetic hyperthermia.

However, the efficiency and effectiveness of the SPMHT method depends very much on the size (diameter (D)) of Fe_3_O_4_ nanoparticles, which is a critical parameter. In addition to this parameter, the shape and distribution of nanoparticles are important, as well as the dipole magnetic interactions when they exist, as well as the intrinsic magnetic observables characteristic of the magnetic material. Also, the parameters of the external magnetic field (AMF) are very important, respectively the amplitude (H) and frequency (f) of the magnetic field, in which the magnetite nanoparticles are found, to produce the hyperthermic effect (heating them to 42–43 °C) necessary in magnetic hyperthermia for destruction of tumor cells. There are many reported experiments with magnetic hyperthermia where the size (diameter D) of the nanoparticles was not properly chosen. Also, the amplitude of the magnetic field H and the values of the frequencies f of the AMF (harmonic) used were not the best suited to obtain the maximum hyperthermic effect in magnetic hyperthermia, therefore the reported results were poor.

Thus, given that SPMHT is more suitable for obtaining the hyperthermic effect in magnetic nanoparticles, and that there are many experimental studies in the field of magnetic hyperthermia with Fe_3_O_4_ nanoparticles, but the nanoparticles used were not at the correct size, although this is a critical parameter, or given the use of inappropriate parameters for the magnetic field (amplitude, frequency), which led to poor results in the application of magnetic hyperthermia. In addition, the lack of a systematic study in the field for optimal conditions in which to obtain the maximum specific loss power and the efficient heating of the nanoparticles, this paper comes to complete that lack and to focus on a systematic study in the filed using a powerful 3D tool. Establishing optimal conditions in SPMHT for nanoparticle size and AMF parameters are key observables in making SPMHT efficient and effective in destroying tumor cells (SPMHT optimization) without affecting healthy tissues. 

The aim of this study is precisely to find the optimal parameters in SPMHT, regarding the optimal diameter of magnetic nanoparticles (*D*_Mop_), the optimal amplitude of the AMF (*H*_op_) applied from the outside and the optimal limit frequency (f_lop_) that can be used in magnetic hyperthermia to obtain maximum efficiency in SPMHT, by reaching the maximum specific loss power (*P*_Mop_) and, at the same time, the maximum efficiency in destroying tumor cells by increasing the temperature (Δ*T*) of magnetic nanoparticles to the one required in hyperthermia of 42–43 °C (Δ*T*_op_) in a short period of time. Having this in view, a 3D computational study was done taking into account the allowable biological limit, in order to efficiently apply SPMHT in vitro, in vivo experiments and later in clinical trials in optimal conditions, for maximum efficacy.

## 2. Theoretical Considerations

The magnetization (*M*) of superparamagnetic nanoparticles in an external magnetic field (*H*) in the absence of interactions between them (dispersed nanoparticle system) is done according to the Langevin function [63,64,65]

(1) $$M=M_{sat}\left(\coth\zeta -\frac{1}{\zeta }\right),$$

where the parenthesis is the Langevin function L(ζ) as in the case of paramagnetic atomic systems [63], $M_{sat}$ is the saturation magnetization of the nanoparticles [64], and *ζ* is the argument of the Langevin function, given by the following relation

(2) $$\zeta =\frac{\mu_{0}m_{NP}H}{k_{B}T}.$$

In this formula $m_{NP}$ is the magnetic moment of the nanoparticle [64], *T* is temperature, $\mu_{0}$ is the magnetic permeability of the vacuum ($4\pi \;\times \;10^{-7}\;H/m$) and $k_{B}$ is the Boltzmann’s constant ($1.38\;\times \;10^{-23}J/K$). 

The saturation magnetization of the monodispersed nanoparticles is

(3) $$M_{sat}=nm_{NP},$$

where n is the concentration of nanoparticles, and $m_{NP}$ is the magnetic moment of the nanoparticle given by formula [66]

(4) $$m_{NP}=V_{NP}M_{s}.$$

In this relation $M_{s}$ is the spontaneous magnetization of a nanoparticle and $V_{NP}$ is the volume of the nanoparticle.

In the approximation of spherical nanoparticles the magnetic moment can be expressed as a function of the diameter of the nanoparticle *D* as

(5) $$m_{NP}=\frac{\pi D^{3}M_{s}}{6}.$$

For low amplitudes of the magnetic field H, when the condition ζ≪1 [67] is fulfilled, the Langevin function develops in series obtaining the expression

(6) $$L\left(\zeta \right)=\frac{1}{3}\zeta +\ldots$$

because

(7) $$\coth\zeta =\frac{1}{\zeta }+\frac{1}{3}\zeta +\ldots$$

Replacing the Langevin function (Equation (6)) in Equation (1), taking into account the Equation (3), the magnetization of the nanoparticle system in low fields is obtained,

(8) $$M_{H\ll }=\frac{nm_{NP}\zeta \;}{3},$$

and

(9) $$M_{H\ll }=\frac{\mu_{0}m_{NP}M_{sat}H}{3k_{B}T}.$$

respectively, taking into account Equation (2).

Considering the magnetic volume fraction in the case of dispersed magnetic nanoparticles $\varepsilon =M_{sat}/M_{s}$ [68], the magnetization is finally obtained.

(10) $$M_{H\ll }=\frac{\varepsilon \mu_{0}M_{s}^{2}V_{NP}H}{3k_{B}T},$$

which allows the determination of the initial magnetic susceptibility of the magnetic nanoparticle system. Under these conditions, the initial magnetic susceptibility $\chi_{i}=\;M_{H\ll }/H$ is obtained,

(11) $$\chi_{i}=\frac{\varepsilon \pi \mu_{0}M_{s}^{2}D^{3}}{18k_{B}T},$$

which is a constant for the given parameters ε, T, and D. 

In an alternating magnetic field (AMF) with a frequency of the order of hundreds of kHz the magnetic nanoparticles heat up, and the volume loss power (power density) is given by the relation [2,32]

(12) $$p=\pi \mu_{0}\chi^{\prime \prime }fH^{2},$$

where *H* is the amplitude and *f* is the frequency of AMF, and $\chi^{\prime \prime }$ is the component of the imaginary part of the complex magnetic susceptibility

(13) $$\chi =\chi^{\prime }-j\chi^{\prime \prime }.$$

In the case of magnetic relaxation the susceptibility $\chi^{\prime \prime }$ is given by formula [69]

(14) $$\chi^{\prime \prime }=\chi_{0}\frac{\omega \tau }{1+{\left(\omega \tau \right)}^{2}},$$

where $\chi_{0}$ is the static magnetic susceptibility, ω is the pulsation of the alternating magnetic field, ω=2πf, and τ is the magnetic relaxation time.

According to Neel’s theory the magnetic relaxation time (Néel) has the expression [30,70]

(15) $$\tau =\tau_{0}exp\left(\frac{KV_{NP}}{k_{B}T}\right),$$

where *K* is the magnetic anisotropy constant, and $\tau_{0}$ a time constant which usually has the value $10^{-9}\;s$ [71]. For spherical nanoparticles Equation (15) expressed as a function of the nanoparticle diameter (*D*) is:

(16) $$\tau =\tau_{0}exp\left(\frac{\pi KD^{3}}{6k_{B}T}\right).$$

In magnetic hyperthermia the specific loss power $P_{s}=p/m$ is used, which does not depend on the mass (*m*) of the heating magnetic nanoparticles system. In low magnetic fields the static magnetic susceptibility $\chi_{0}$ will be given by the initial magnetic susceptibility: $\chi_{0}\equiv \chi_{i}$. Thus, using Equations (12), (14) and (16), we may finally write the mathematical expression of the specific loss power $P_{s}$ in the case of magnetic nanoparticles in AMF with frequency f and amplitude H:

(17) $$P_{s}=\frac{\pi \mu_{0}\chi_{i}}{\rho }\frac{2\pi f\tau }{1+{\left(2\pi f\tau \right)}^{2}}fH^{2},$$

where

$$\chi_{i}=\frac{\varepsilon \pi \mu_{0}M_{s}^{2}D^{3}}{18k_{B}T}\;,\;\tau =\tau_{0}exp\left(\frac{\pi KD^{3}}{6k_{B}T}\right)\;,$$

are the initial magnetic susceptibility ($\chi_{i}$) given by Equation (11) and the relaxation time (τ) given by Equation (16), expressed according to the diameter *D* (size) of the nanoparticles and the magnetic anisotropy constant (Equation (16)) characteristic of the magnetic nanoparticles.

The measure of the heating of magnetic nanoparticles ($\Delta T_{i}$) is given by the heating rate $\Delta T_{i}/\Delta t$ [72], expressed as a function of the specific loss power in magnetic nanoparticles $P_{s}$ (or the specific absorption rate $SAR=c\Delta T_{i}/\Delta t$ for an adiabatic system),

(18) $$\Delta T_{i}=\frac{P_{s}}{c}\Delta t,$$

where c is the specific heat, and Δt is the duration in which the heating takes place.

In conclusion, Equation (17), together with Equations (11) and (16), and Equation (18) are used in our 3D study to calculate the specific loss power ($P_{s}$) and heating of nanoparticles ($\Delta T_{i}$) in case of low magnetic fields for monodisperse Fe_3_O_4_ nanoparticles (with spontaneous magnetization *M*_s_ and constant of magnetic anisotropy *K*) as a function of the variables: the diameter of the magnetic nanoparticles *D*, the amplitude of the magnetic field *H*, and the frequency *f* of AMF.

## 3. Results and Discutions

### 3.1. Magnetic Nanoparticles and Study Parameters

Our study on the specific loss power in SPMHT, which leads to the heating of magnetic nanoparticles, was done for approximately spherical Fe_3_O_4_ (magnetite) nanoparticles which are the most commonly used in magnetic and superparamagnetic hyperthermia, having the characteristic observables given in Table 1. Also, the table gives the value ranges for the parameters of the alternating magnetic field (AMF), the amplitude (*H*) and the frequency (*f*) of the field considered for this study. All these values were established taking into account the real conditions in which the magnetic hyperthermia experiments are performed, based on the previous results.

A professional software was used for the 3D study and calculation of the observables of interest in SPMHT, and a 3D graphical representation of the results was generated. The 3D representation allows observation at the same time of the dependence of the observables of interest as a function of two variables, thus providing more information than in a simple 2D representation for a function of a single variable. Also, the possibility of studying for a wide range of values allows an overview of the variation of the respective observables, so that the optimal values can be extracted. Thus, the 3D representation is a powerful tool for a much more complete study.

The observables pursued in our study, which are of particular interest in the efficient practical application of SMHT, are the specific loss power ($P_{s}$) (Equation (17) with Equations (11) and (16)) and the heating temperature (ΔT) (Equation (18)) of magnetic nanoparticles after the application of AMF. Our study of SPMHT optimization for the efficient application of the method in hyperthermia experiments such as in vitro, in vivo and in clinical trials, were made as a function of the diameter of Fe_3_O_4_ nanoparticles (*D*), which is a critical parameter in SPMHT, as well as the amplitude of magnetic field (*H*) and the frequency of AMF (*f*). For the magnetic volume fraction (*ε*), the usual values in the case of magnetic nanoparticle dispersions were used. The study was done at room temperature, considering the temperature value of 298 K.

### 3.2. The Specific Loss Power for Low Magnetic Fields

The observables that indicate whether the magnetic nanoparticles are suitable for use in SPMHT or not, in terms of their heating in an alternating magnetic field with a frequency of hundreds of kHz, are the specific loss power (*P_s_*) given by Equation (17) (or specific absorption rate (SAR) for an adiabatic system), and then the heating rate (ΔT/Δt) of nanoparticles given by Equation (18). Based on the previous experimental results [2,3,6,8,10], the specific loss power must have values of at least tens of W/g, in order to be able to lead to the necessary heating level in magnetic hyperthermia, respectively to a temperature of 42–43 °C, which is necessary for the destruction of tumor cells by apoptosis.

**Figure 1:**
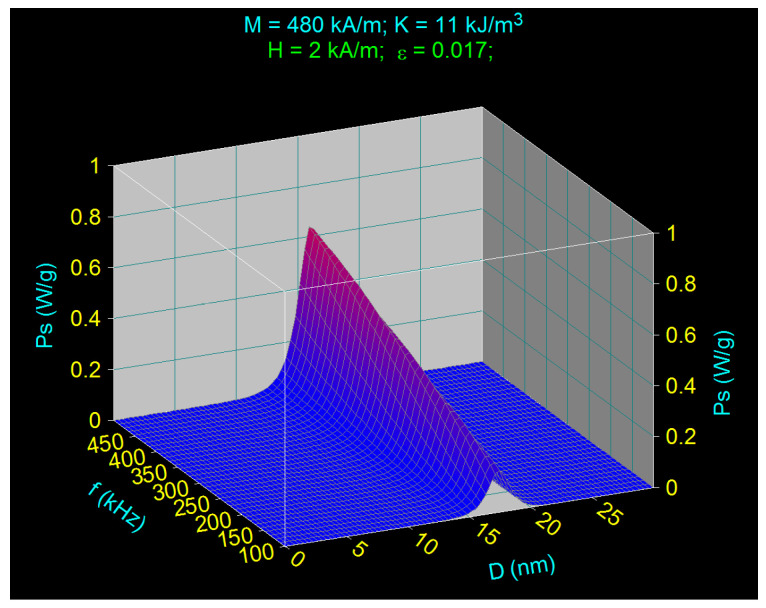
The specific loss power as a function of the diameter of magnetic nanoparticles and the AMF frequency for the magnetic field amplitude of 2 kA/m.

In agreement with the theory presented in Section 2, in order that the power *P_s_* is correctly calculated it is necessary that the amplitude of the AMF is low, so that the condition given by Equation (6) can be fulfilled. Calculating the power *P_s_* under these conditions as a function of the nanoparticle diameter *D* and the AMF frequency *f*, *P_s_ = P_s_(D,f)*, considering a field of 2 kA/m, we obtain the result shown in Figure 1. The usual values used in magnetic hyperthermia for the magnetic volume fraction of nanoparticles (0.017) and the AMF frequency (100–500 KHz) were considered in the calculations.

The 3D representation in the figure shows two very important aspects:

(i) The specific loss power *P_s_* critically depends on the diameter *D* of Fe_3_O_4_ nanoparticles, having a high maximum at the values of diameters (*D_M_*) ~16–17 nm, these depending slightly on the frequency (*D_M_* diameter decreases slightly with increasing frequency). For diameters *D* less than or greater than *D_M_*, respectively *D* less than ~15 nm at 100 kHz and ~13 nm at 500 kHz, and for *D* greater than ~20 nm at 100 kHz and ~18 nm at 500 kHz, the specific loss power becomes almost zero. This variation of specific loss power shows that it strongly depends on the diameter of the nanoparticles, and that the diameter of the nanoparticle is a critical parameter, *P_s_* having a maximum value (*P_sM_*) at the diameter of *D_M_*. Also, the maximum specific loss power *P_sM_* increases approximately in proportion to the increase in AMF frequency. This result clearly shows that in SPMHT the nanoparticles cannot be of any size, because at larger or smaller nanoparticle sizes than *D_M_*, the specific loss power decreases rapidly to zero, and the hyperthermic effect can no longer be obtained. In other words, before starting any experiment with magnetic hyperthermia, the first thing to do is to determine the value of the critical diameter *D_M_* (critical size) of the nanoparticles, which will lead to a maximum dissipated power, and as an effect, to an efficient heating of magnetic nanoparticles. Otherwise, for other values of the diameter (sizes) of the nanoparticles the hyperthermic effect will be weak or will even be lacking. In the scientific literature are reported many experiments of magnetic hyperthermia where inappropriate diameters (sizes) of magnetic nanoparticles have been used, which have led to poor results [4,21,22,55,60].
(ii) In low magnetic fields, although Equation (6) is well satisfied, the maximum specific loss power at the critical diameter is very small, so that under these conditions, the heating of the nanoparticles will also be reduced and inefficient in magnetic hyperthermia of tumors. For the case presented even at the highest frequency of 500 kHz the *P_sM_* power will be quite low, below the value of 0.7 W/g, a value that is unsuitable for magnetic hyperthermia.

Thus, in order to increase the efficiency in magnetic hyperthermia a high incease of the maximum specific loss power *P_sM_* is needed, that will lead to an efficient heating of magnetic nanoparticles with *D_M_* diameter, so that the temperature of 42–43 °C required in hyperthermia can be reached, and also, in a short time, so as not to affect healthy cells. For the fixed parameters of the magnetic nanoparticles the increase of the maximum power *P_sM_* for a *D_M_* diameter can be obtained efficiently by increasing the *H* amplitude of magnetic field and/or the frequency *f* of the AMF. Also, another parameter is *ε*, but this is limited to maximum values of up to 0.15 in the case of magnetic nanoparticles. 

**Figure 2:**
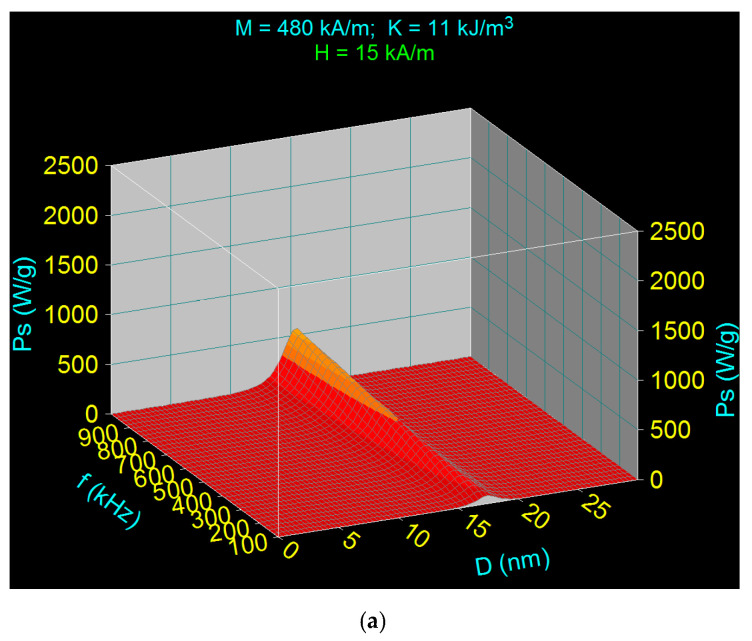

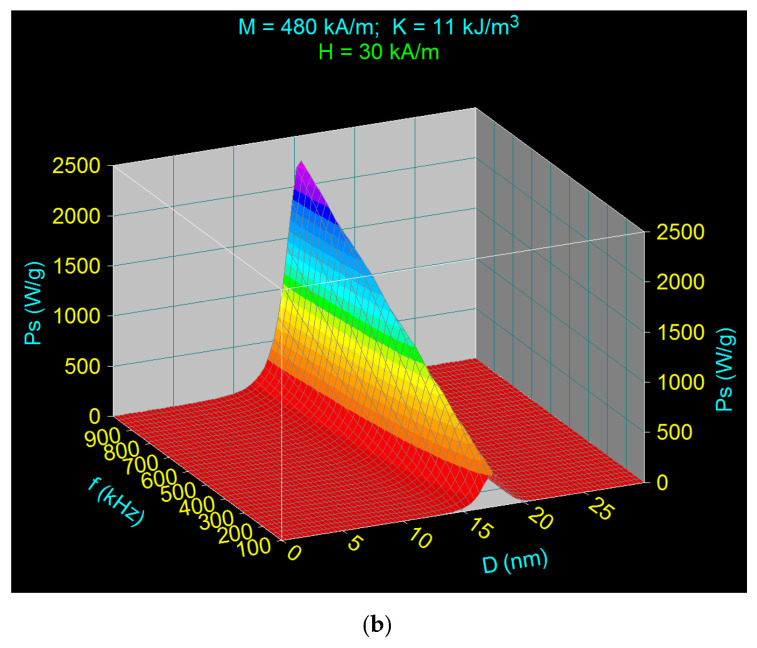
The specific loss power variation according to the Equation (17) depends on the nanoparticle diameter and the AMF frequency for the magnetic field amplitude of (**a**) 15 kA/m and (**b**) 30 kA/m.

**Figure 3:**
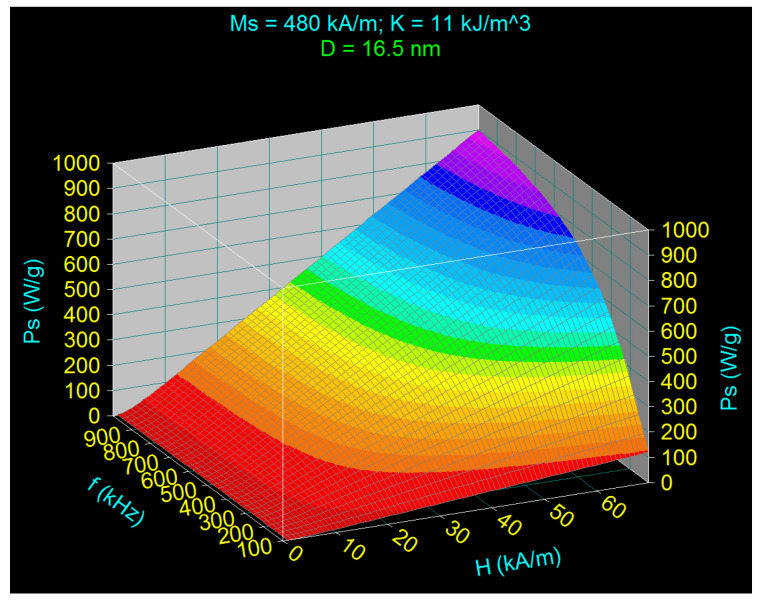
Maximum specific loss power depending on the amplitude and frequency of the AMF, for the diameter of the nanoparticle corresponding to the maximum power.

Figure 2 shows the cases when the frequency f increases from 100 to 1000 kHz and the magnetic field *H* increases from 15 kA/m (a) to 30 kA/m (b), considering for *ε* the value of 0.15. According to the obtained results, and using Equation (17), results are more efficient to increase the maximum specific loss power by increasing the amplitude of magnetic field (Figure 2b) and not the frequency, considering also the dependence of the power on the square of the magnetic field. This aspect is clearly shown in Figure 3, where the maximum specific loss power (*P_sM_*) obtained was represented for *D_M_* of 16.5 nm as a function of the amplitude of magnetic field in the range 1–70 kA/m. Furthermore, from a practical and technical point of view it is easier to obtain larger magnetic fields at lower frequencies than large fields at high frequencies.

However, for in vitro, in vivo, and in clinical trials magnetic hyperthermia experiments, the biological limitation for the magnetic field and the frequency AMF imposed by the following formula [73]

(19) $$H\;\cdot \;f<5\;\cdot \;10^{9}\;\left(Am^{-1}Hz\right)$$

must be taken into account in order not to affect the healthy tissues. Thus, Table 2 shows the limit values for the frequency (*f_l_*) AMF depending on the values established for the magnetic field in the range 5–50 kA/m, and the pairs of values for (*H,f_l_*), respectively that can be used in the range 100–1000 kHz for the limit value *H × f_l_* given by Equation (19).

However, the use of a large magnetic field, although advantageous in terms of dissipated power, means that Formula (17) for calculating the specific loss power under linear conditions of magnetization of nanoparticles in low amplitude of AMF can no longer be used. This formula must be modified taking into account the magnetization of nanoparticles in the large field. But in these conditions the questions naturally arise: (a) how will the large magnetic fields affect the maximum dissipated power? (b) can high values for the magnetic field be used in practice? The answers to these questions will be given in the next section.

### 3.3. The Specific Loss Power for Large Magnetic Fields

When applying a high amplitude AMF (greater than a few kA/m) the magnetization of the nanoparticles will no longer be linear [74], but will follow the Langevin function in large fields [65], and in these conditions the approximation given by Equation (6) can no longer be used. Thus, the magnetic susceptibility in the formula of the specific loss power (*P_s_*) (Equation (17)) will no longer be a constant, and equal to the initial magnetic susceptibility, but will be given (by definition $\chi_{0}=M/H$) by the formula

(20) $$\chi_{0}=\frac{M_{sat}}{H}\left(\coth\zeta -\frac{1}{\zeta }\right)\;.$$

So, in this case when a large amplitude magnetic field is applied the susceptibility will be a nonlinear magnetic field function (Equation (2)).

Expressing the static magnetic susceptibility ($\chi_{0}$) as a function of the initial magnetic susceptibility ($\chi_{i}$) given by Equation (11), and taking into account the Equations (4) and (8), is obtained

(21) $$\chi_{0}=\frac{3\chi_{i}}{\zeta }\left(\coth\zeta -\frac{1}{\zeta }\right)\;.$$

Thus, the specific loss power in the case of large amplitude magnetic fields, according to Equations (12), (14), (16) and (21), will have the formula:

(22) $$P_{s}=\frac{3\pi \mu_{0}\chi_{i}}{\rho \zeta }\left(\coth\zeta -\frac{1}{\zeta }\right)\frac{2\pi f\tau }{1+{\left(2\pi f\tau \right)}^{2}}fH^{2}.$$

where

$$\chi_{i}=\frac{\varepsilon \pi \mu_{0}M_{s}^{2}D^{3}}{18k_{B}T}\;,\;\tau =\tau_{0}exp\left(\frac{\pi KD^{3}}{6k_{B}T}\right),\;\zeta =\frac{\pi \mu_{0}M_{s}D^{3}}{6k_{B}T}H,$$

are given by Equations (2), (5), (11) and (16) expressed as a function of nanoparticle diameter *D*.

In this case the calculations for the specific loss power will be made using Equation (22), with $\chi_{i}$ given by Equation (11), and not Equation (17), because the static magnetic susceptibility $\chi_{0}$ is now a function of the amplitude of magnetic field *H* (Equation (2)). Thus, the heating temperature in this case will be given by the same Formula (18), but with P_s_ given by the new Equation (22).

**Figure 4:**
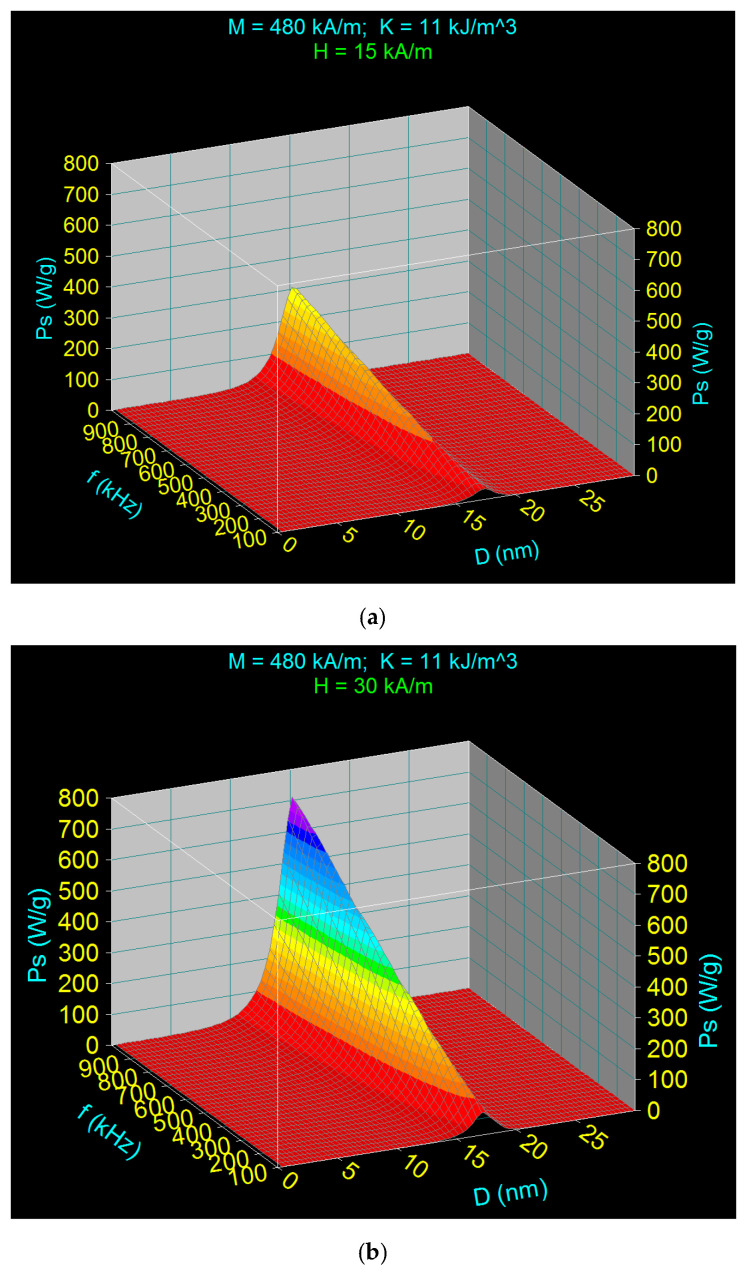
The specific loss power variation according to the Formula (22) depending on the nanoparticle diameter and the AMF frequency for the magnetic field amplitude of (**a**) 15 kA/m and (**b**) 30 kA/m.

Doing the calculations in the same conditions as in the previous case shown in Figure 2a,b, the results in Figure 4 are obtained for the magnetic field of 15 kA/m (Figure 4a) and 30 kA/m (Figure 4b). Comparing the results from Figure 4 with those from Figure 2, a significant decrease in the maximum specific loss power *P_sM_* can be observed, so at the frequency (maximum) of 1000 kHz and *D_M_* ~15 nm the maximum for *P_s_* decreases from ~500 W/g to ~300 W/g for the field of 15 kA/m, and from ~2125 W/g to ~700 W/g for the field of 30 kA/m. At the same time, there is also a greater decrease of the maximum specific loss power (*P_sM_*) when the magnetic field has a higher value of 30 kA/m, respectively.

**Figure 5:**
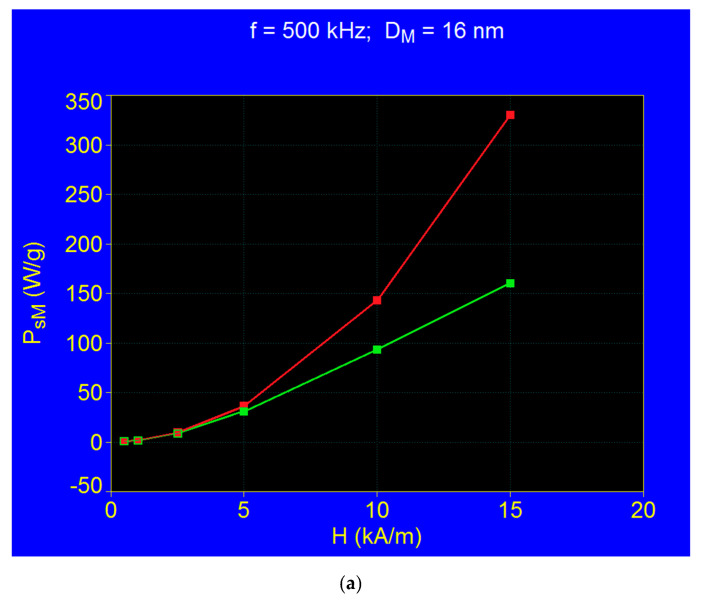

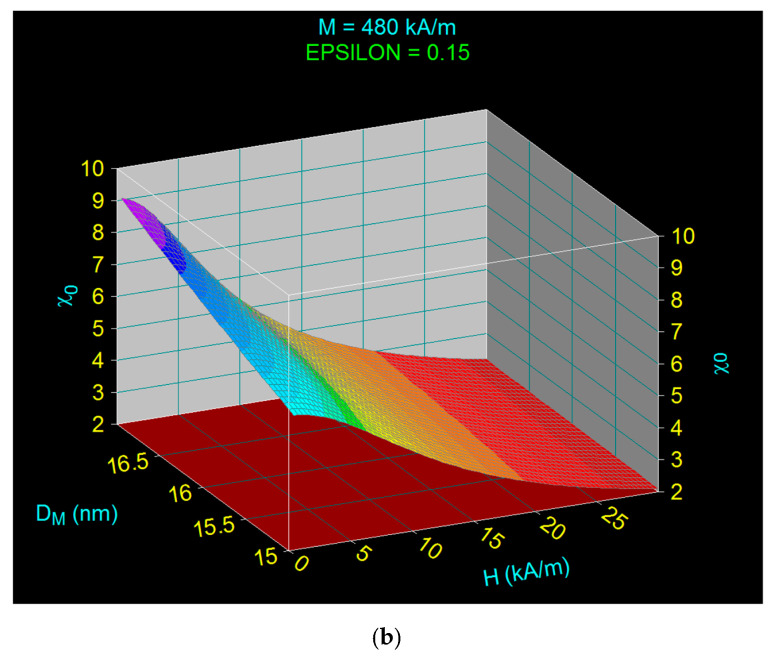
(**a**) Maximum specific loss power (*P_sM_*) variation as a function of the amplitude of magnetic field *H*, calculated by Equation (17) (red curve) and Equation (22) (green curve), for nanoparticle diameter of 16 nm (corresponding to maximum power) and AMF frequency of 500 kHz; (**b**) The variation of the static magnetic susceptibility with the magnetic field for different values of the diameter of magnetic nanoparticles that give the maximum specific loss power.

Using the formulas given by Equation (17), and then Equation (22) the specific loss powers *P_s_* were calculated in both cases for the values of the magnetic field of 0.5; 1; 2.5; 5; 10; and 15 kA/m (as in Figure 2 and Figure 4). The calculations were made in both cases for the same frequency of 500 kHz and the same critical diameter of the nanoparticles *D_M_* ~16 nm that gives the maximum specific loss power (*P_sM_*). From the curves thus obtained in both cases, for each value of the magnetic field the maximum values of the specific loss power *P_sM_*, were extracted, obtaining the curves from Figure 5a. The red curve in the figure corresponds to the case when the magnetic susceptibility $\chi_{0}$ is considered constant and equal to the initial one $\chi_{i}$, and the green curve corresponds to the case when the magnetic susceptibility is no longer constant, but depends on the magnetic field at large values (in the figure for *H* > 5 kA/m). At low values of the magnetic field, up to 2.5 kA/m, the powers in both cases are the same (Figure 5a), and the formula given by Equation (22) is reduced in this case to the formula given by Equation (17). Significant differences begin to occur for magnetic fields greater than 5 kA/m, when the maximum specific loss power follows the green curve instead of the red curve. Thus, the values of the power on the red curve for *H* greater than 5 kA/m do not correspond to physical reality, and cannot be taken into account, since they were determined considering magnetic susceptibility constant at large fields, which in reality is not the case. 

In large magnetic fields, static magnetic susceptibility $\chi_{0}$ is a function of the field amplitude, and is not a constant. Figure 5b shows the variation of the magnetic susceptibility $\chi_{0}$ when the amplitude of the magnetic field increases up to 30 kA/m, for different values of the *D_M_* diameter in the range 15–17 nm (values that correspond to the maximum specific loss power specifically). Thus, as seen in Figure 5b, the decrease in maximum specific loss power *P_sM_* with increasing magnetic field over 5 kA/m is due to decreasing magnetic susceptibility with increasing AMF amplitude.

To conclude, the realistic formula for calculating the specific loss power at large magnetic fields is that given by Equation (22). This is reduced to the formula given by Equation (17) only for low magnetic fields.

However, in a high magnetic field another important aspect from the point of view of SPMHT is the following: what is the maximum value for the magnetic field that can be used? At first appearance, from the point of view of magnetic hyperthermia (maximum specific loss power) according to the data in Table 2 and the results from Figure 4 and Figure 5a (green curve) and Equation (22), it seems that it would be more advantageous to use high magnetic fields in SPMHT. According to Equation (19) for the biological limit and the values in Table 2 for high magnetic fields, the result is that even a magnetic field of 100 kA/m could be used at the limit (maximum) frequency of 50 kHz. However, is such an approach realistic? This issue will be clarified in the next section.

### 3.4. Optimization in Superparamagnetic Hyperthermia: The Optimal Maximum Specific Loss Power

Calculating the maximum specific loss power using Equation (22), from its 3D representation as in Figure 4, for the values of magnetic field *H* and frequency f in Table 2, the values of the maximum specific loss power given in Table 3 were found. In the table are also given the values of the diameters of the magnetite nanoparticles for which the maximum specific loss powers are obtained at the values of the limit frequencies.

Having in view the results obtained in Table 3 for the maximum specific loss power *P_sM_* and the diameters *D_M_* that the nanoparticles must have to obtain the maximum power, the following question arises: what are the optimal values for the magnetic field, frequency and diameter of magnetic nanoparticles, within the biological permissible limit, in order to obtain the optimal maximum for specific loss power (*P_sMop_*) for the efficient heating of the magnetic nanoparticles, and finally for obtaining the optimal temperature required in SPMHT, of 42–43 °C, in the shortest time, so as not to affect the healing tissues. For a practical implementation of the SPMHT method it is essential to know all these aspects so that the method can be used effectively and with maximum efficiency in the destruction of tumor cells. Outside the optimal conditions poor results will be obtained in the superparamagnetic hyperthermia of the tumors, depending on the values that the parameters *D*, *H* and *f* will have compared to the optimal ones.

**Figure 6:**
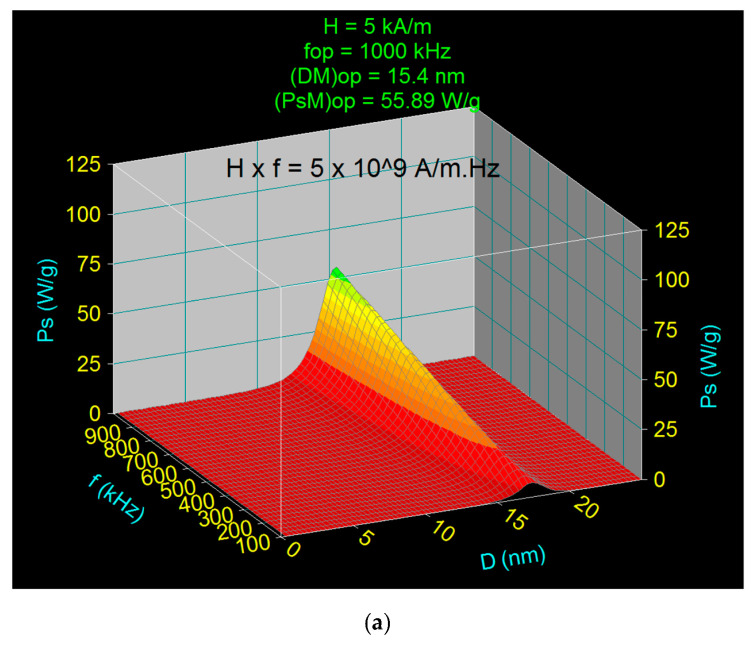

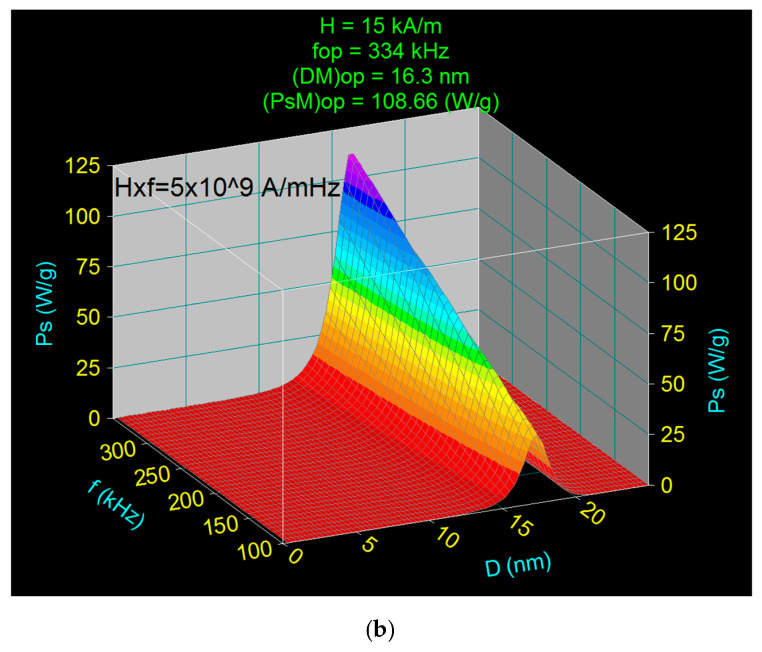
Optimal specific loss power within the allowable biological limit, for the magnetic field amplitude of (**a**) 5 kA/m and (**b**) 15 kA/m.

In order to determine the optimal values of the nanoparticle diameter (*D_Mop_*) and the optimal limit frequency (f_lop_) for different values of the magnetic field in the range considered for analysis, 5–50 kA/m (Table 3), leading to the maximum optimal specific power (*P_sMop_*) for superparamagnetic hyperthermia, and finally to determine the optimal heating of the nanoparticles and obtaining the optimal temperature necessary for the destruction of the tumor cells, without affecting the healthy cells around them, the 3D representations of the specific loss power Ps were used, as in Figure 6, for all magnetic field values. The figure shows the 3D representations for the magnetic field of (a) 5 kA/m and (b) 15 kA/m, at the corresponding limit frequencies of (a) 1000 KHz and (b) 334 kHz, from which the values for *D_Mop_* and *P_sMop_* were determined.

**Figure 7:**
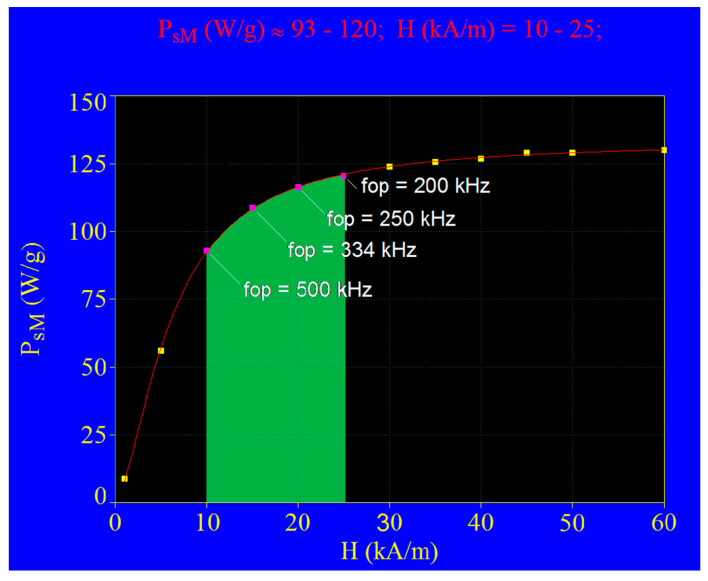
The maximum specific loss power variation with the amplitude of magnetic field within the biologically admissible limit; fop is the optimal limit frequency that gives the highest power depending on the magnetic field.

From all 3D representations as in Figure 6 with the values in Table 3 for the magnetic field, all *P_sM_* and *D_M_* values were determined at the corresponding limit frequencies (*f_l_*). With the obtained data, the curve representing *P_sM_* as a function of the magnetic field H from Figure 7 was determined.

From the obtained results (Figure 7) some very important aspects are remarked.

(1) Not all values for the magnetic field and the frequency limit can be used to obtain an optimal specific loss power: for example, a power high enough to efficiently heat magnetic nanoparticles, but not to use unnecessary resources in the production of the magnetic field. Thus, from the variation of the maximum power specifically it results at magnetic fields higher than 25–30 kA/m, for the limit values of the AMF frequency specified in Table 3, the power increase is very low, and at values higher than 40 kA/m practically it reaches a saturation of power, when further increase of the magnetic field no longer determines any increase of the specific loss power. Thus, magnetic fields higher than 40 kA/m cannot be used to increase the specific loss power, and the temperature, in the case of magnetite nanoparticles with a diameter of ~17 nm, because they no longer have any effect on the increase in power. The same remark is valid for field values between 25–40 kA/m, since the increase of power is insignificant in the considered interval (Figure 7).
(2) For magnetic fields lower than 10 kA/m, although the specific loss power increases rapidly with increasing magnetic field, it will still have lower values than in the range 10–25/30 kA/m. In addition to this, it is also very important that for low fields below 10 kA/m the frequency limit AMF must increase from 500 kHz to over 1000 kHz, which raises possible problems of affecting healthy tissues at high frequencies, especially at over 1000 kHz. It is also technically more difficult to obtain the required magnetic fields at frequencies higher than 500 kHz. In addition, the power being significantly reduced in this interval will not obtain an efficient (fast) heating of the nanoparticles.

In conclusion, considering these results, the recommended interval for the optimal maximum specific loss power (*P_sMop_*), and for the efficient practical application of SPMHT with Fe_3_O_4_ nanoparticles, is the one included in the magnetic field range of 10–25 kA/m, delimited in Figure 7 by green area and in Table 3 by green values for the optimal magnetic field *H_op_* and the optimal limit frequency (*f_lop_*) 200–500 kHz. The optimal *D_Mop_* diameter of the nanoparticles, corresponding to each pair (*Hf*)*_op_* are shown in green color in the table. Example, for the magnetic field of 15 kA/m (*H_op_*) the applicable optimal limit frequency (*f_lop_*) will be 334 kHz, and the Fe_3_O_4_ nanoparticles must have the optimal diameter (*D_Mop_*) of 16.3 nm in which case an optimal specific loss power (*P_sMop_*) is obtained of 108.66 W/g, which will lead to the optimal, efficient/fast heating of the nanoparticles, these reaching in a very short time the value of the temperature used in magnetic hyperthermia (42–43 °C). In the next section this issue will be presented and discussed.

At the end of this section, we must also mention the fact that at the field value of 15 kA/m, within the admissible biological limit, the power *P_sMop_* is significantly lower (being of ~108 W/g) compared to the case shown in Figure 4a, where the power reaches ~300 W/g. However, the frequency used in the case shown in Figure 4a exceeds the permissible biological limit, and therefore cannot be a used in SPMHT. Thus, the maximum value of power that can be obtained within the allowable biological limit is 108.66 W/g. Obviously, powers lower than 108.66 W/g can be used in practice, or the optimal values of the *P_sMop_* power shown in Figure 7 (rose points) and Table 3 (green values), using frequencies lower than the optimal limit value, but in these cases the heating efficiency will be reduced.

### 3.5. Efficient Heating of Magnetic Nanoparticles

**Figure 8:**
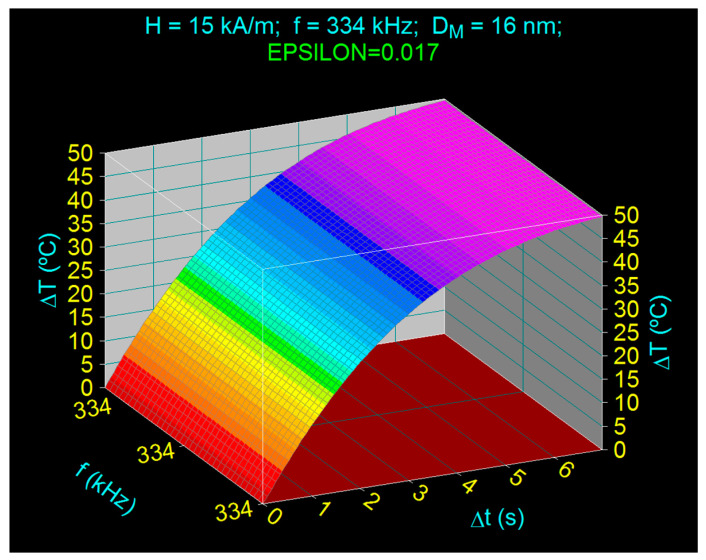
The time variation of the heating temperature of the magnetic nanoparticles in the optimal conditions (when the diameter of the nanoparticles is 16 nm, the magnetic field is 15 kA/m and the frequency of AMF in the admissible biological limit is 334 kHz).

Using the specific loss power given by Equation (22) for large magnetic field and then Equation (18) for the heating rate, we calculated the heating temperature of Fe_3_O_4_ nanoparticles in the optimal conditions previously determined, for the magnetic field in the optimal region of 15 kA/m, the optimal *f_lop_* frequency limit of 334 kHz and the optimal diameter *D_M_* of ~16 nm of the nanoparticles, which gives the optimal maximum of the specific loss power (*P_sMop_*). In the calculations the unfavorable case used in practice, when *ε* is 0.017, was considered for the magnetic volume fraction in order to see if an efficient heating can be obtained even in these conditions. Also, the calculations took into account the correction brought to the temperature in the Equations (2), (11) and (16), when it increases above the room temperature of 298 K. The obtained result is shown in Figure 8, where the rapid and efficient increase of the temperature is very clearly seen, this reaching the value of 50 °C in a very short time of only 7 s, and the one necessary in SPMHT of the tumors, of 42–43 °C, in only 4.1–4.3 s. From the point of view of the practical application of magnetic hyperthermia this result is very important, as the duration of application of the AMF field is shortened to reach the required temperature in SPMHT, thus healthy cells are not affected.

The obtained result confirms the correct approach of our optimization in SPMHT by establishing the optimal parameters, so that SPMHT can be applied efficiently in hyperthermia experiments to obtain the maximum effect: efficient heating at 42–43 °C in a short time.

Following our optimization study the optimal parameters required in suparparamagnetic hypertermia, the *D_Mop_* diameter of the nanoparticles, the *H_op_* magnetic field amplitude, the optimal *f_lop_* limit frequency of the AMF, and the *ε* volume fraction of the nanoparticles can be correctly established for the efficient application of SPMHT in vitro, in vivo, and in clinical trials, with maximum efficacy on tumors.

Other relevant previous computational studies [32,72,74,75,76] do not establish the optimal conditions for the practical implementation of SPMHT using magnetite nanoparticles, as it is in our case, by correctly establishing the size (diameter) that the nanoparticles must have (*D_Mop_* ~16 nm), the values of the optimal amplitude and frequency of AMF (*H_op_* and *f_lop_*) to obtain the highest maximum of the specific loss power (*P_sMop_*) within the admissible biological limit. In Refs. [32,72,75] presents a general 2D study on the heating rate (Δ*t*/Δ*t*) depending on different parameters: radius particles [32,72], viscosity and dispersion of nanoparticles in a ferrofluid [32] or temperature [75]. Also, in Ref. [32] indicates the value of the diameter of the magnetite nanoparticles <14 nm which leads to the maximum heating rate, and in Ref. [76] the diameter is 12 nm which leads to the maximum SAR (specific absorption rate), a diameter which is underestimated, probably due to the consideration in theory of a higher anisotropy constant for magnetite than the real one [34,35]. An interesting computational study on SAR (SLP or *P_s_* in our case, respectively) for a model with cubic AC susceptibility in the nonlinear regime was recently presented in Ref. [74], but it shows the effect that a DC magnetic field and interactions can have on SAR optimization in magnetic nanoparticles, within the allowable biological limit.

In addition, in our case the presentation of the direct dependence of heating temperature (Δ*T_i_*) in time, where the aim is to reach the temperature of 43 °C required in tumor hyperthermia, is much more suggestive than the heating rate for the practical application of SPMHT. Also, by using the 3D power tool, compared to other previous 2D studies, it is possible to know the specific loss power dependence simultaneously by two variables of high interest in SPMHT such as nanoparticle size and frequency of AMF, and this for a very wide range of values which allows the capture of other variations of finesse that in 2D can go unnoticed. Thus, our study will obtain more complete information on the observables and processes that contribute to the increase of specific loss power, so that the optimization is done accordingly in order to obtain the maximum effect in SPMHT when implementing this technique.

Compared to some relevant experimental data that have given good results in vivo cancer therapy and in clinical trials, our theoretical results are in good agreement. Thus, regarding the size of Fe_3_O_4_ nanoparticles (*D_M_* ~16 nm) that leads to the maximum effect in magnetic hyperthermia, it is in good agreement with the one already used in magnetic hyperthermia experiments in clinical trials where used nanoparticles with a diameter of 15 nm [9,10,11,12,14]. The same size is currently used in NanoTherm^®^ clinical therapy approved in Germany (Berlin) for treating brain tumors using magnetic hyperthermia [77]. Also, the values in the range determined by us for the magnetic field amplitude and/or frequency of AMF that are optimal in magnetic hyperthermia, within the allowable biological limit, have been used in various magnetic hyperthermia experiments that have given good results in tumor therapy in vitro, in vivo and clinical trials [8,9,11,14,18,46,78,79]. The agreement of our theoretical results with the experimental ones regarding the implementation of magnetic hyperthermia demonstrates the validity of our theoretical results.

In addition, by considering the optimal conditions and parameters determined by us, it will certainly increase the effectiveness in treating cancer by magnetic hyperthermia.

## 4. Conclusions

For the efficient practical implementation of SPMHT with ferrimagnetic nanoparticles of F_3_O_4_, with maximum efficacy in tumor therapy, the following optimization conditions in SPMHT must be met simultaneously:

(1) Ferrimagnetic nanoparticles must have the optimal diameter (or the optimal medium diameter) in the range 16.0–16.8 nm, depending on the AMF frequency used in magnetic hyperthermia: 16.0 nm for the frequency of 500 kHz and 16.8 for the frequency of 200 kHz, which leads to a maximum of specific loss power (*P_sM_*);
(2) The optimal magnetic fields used must be in the range of 10–25 kA/m, depending on the power that is required in the superparamagnetic hyperthermia experiment, in which case the specific loss power given by Equation (22) will be used;
(3) The optimal frequency of the AMF must be located in the range of 200–500 kHz, depending on the amplitude of the magnetic field, with the fulfillment of the condition for the admissible biological limit (Equation (19)): 200 kHz for the field of 25 kA/m and 500 kHz for the 10 kA/m field;
(4) Under the above conditions, the maximum optimal specific power, *P_sMop_* (with the values given in Table 3) is obtained, which leads to the rapid and efficient heating of the Fe_3_O_4_ nanoparticles (Figure 8) in SPMHT, depending on the volume fraction of nanoparticles in dispersion;
(5) Under optimal conditions, *D_Mop_* of 16.3 nm, H_op_ of 15 kA/m, optimal limit frequency of 334 kHz, at a volume fraction of 0.017, for Fe_3_O_4_ nanoparticles the heating temperature of 42–43 °C required in hyperthermia for the destruction of tumor cells is obtained in a very short time of only 4.1–4.3 s, with major benefits in the practical implementation of the SPMHT method, in order not to affect the healthy tissues;

The exact values for the optimal parameters, nanoparticle diameter *D_Mop_*, magnetic field amplitude *H_op_* and AMF frequency *f_lop_* in different cases are found in Table 3.

Our study will allow the use of SPMHT in in vitro, in vivo and then in clinical trials experiments of SPMHT in conditions of maximum efficiency in order to obtain the maximum specific loss power, and with maximum efficacy on malignant tumor cells in alternative cancer therapy, by obtaining the necessary temperature in hyperthermia in a very short time.

## Figures and Tables

**Table 1:** The values of characteristic observables of Fe_3_O_4_ nanoparticles and alternating magnetic field (AMF) parameters; *D* is the diameter, *M*_s_ is the spontaneous magnetization, *K* is the anisotropy constant, *ρ* is the material density, *c* is the specific heat, and *ε* is the volumic magnetic fraction.

*D* (nm)	*M_s_* (kA/m)	*K* (kJ/m^3^)	*ρ* (kg/m^3^)	*c* (J/kgK)	*ε*	*H* (kA/m)	*f* (kHz)
5–25	480	11	5.24 × 10^3^	670	0.01–0.15	1–50	100–1000

**Table 2:** Magnetic field and AMF frequency values for the biologically permissible limit.

*H* (kA/m)	5	10	15	20	25	30	35	40	45	50
*f_l_* (kHz)	1000	500	334	250	200	167	143	125	112	100
*H* × *f_l_* (A/m.Hz)	5 × 10^9^	5 × 10^9^	5 × 10^9^	5 × 10^9^	5 × 10^9^	5 × 10^9^	5 × 10^9^	5 × 10^9^	5 × 10^9^	5 × 10^9^

**Table 3:** The values of the maximum specific loss power *P_sM_* determined for the critical diameters *D_M_* in the admissible biological limit ($H\;\times \;f_{l}=5\;\times \;10^{9}\;{\mathrm{Am}}^{-1}\mathrm{Hz}$).

Observables	Values									
*H* (kA/m)	5	10	15	20	25	30	35	40	45	50
*f_l_* (kHz)	1000	500	334	250	200	167	143	125	112	100
*D_M_* (nm)	15.4	16.0	16.3	16.6	16.8	16.9	17.0	17.1	17.2	17.3
*P_sM_* (W/g)	55.89	92.27	108.66	116.10	120.34	123.65	125.45	126.74	128.92	128.79

## Data Availability

This is not applicable.
